# Gastrointestinal bleeding in children: diagnostic approach

**DOI:** 10.1186/s13052-024-01592-2

**Published:** 2024-01-23

**Authors:** Marisa Piccirillo, Valentina Pucinischi, Maurizio Mennini, Caterina Strisciuglio, Elsa Iannicelli, Maria Agostina Giallorenzi, Silvia Furio, Alessandro Ferretti, Pasquale Parisi, Giovanni Di Nardo

**Affiliations:** 1 NESMOS Department, Faculty of Medicine and Psychology, Sapienza University of Rome, Pediatric Unit, Sant’Andrea University Hospital, Via Di Grottarossa1035-1039, 00189 Rome, Italy; 2 Department of Woman, Child and General and Specialistic Surgery, University of Campania “Luigi Vanvitelli”, Naples, Italy; 3 Department of Medical Surgical Sciences and Translational Medicine, Sapienza University of Rome, Sant’Andrea University Hospital, Radiology Unit, Rome, Italy

**Keywords:** Gastrointestinal bleeding, Pediatric, Hematemesis, Hematochezia, Melena, Endoscopy, Abdominal CT, Abdominal US, MR

## Abstract

Different conditions may underlie gastrointestinal bleeding (GIB) in children. The estimated prevalence of GIB in children is 6.4%, with spontaneous resolution in approximately 80% of cases. Nonetheless, the initial approach plays a pivotal role in determining the prognosis. The priority is the stabilization of hemodynamic status, followed by a systematic diagnostic approach. GIB can originate from either upper or lower gastrointestinal tract, leading to a broad differential diagnosis in infants and children. This includes benign and self-limiting disorders, alongside serious conditions necessitating immediate treatment. We performed a nonsystematic review of the literature, in order to describe the variety of conditions responsible for GIB in pediatric patients and to outline diagnostic pathways according to patients’ age, suspected site of bleeding and type of bleeding which can help pediatricians in clinical practice. Diagnostic modalities may include esophagogastroduodenoscopy and colonoscopy, abdominal ultrasonography or computed tomography and, when necessary, magnetic resonance imaging. In this review, we critically assess these procedures, emphasizing their respective advantages and limitations concerning specific clinical scenarios.

## Introduction

Gastrointestinal bleeding (GIB) is a common condition in children, with a reported incidence of 6.4% [1, 2]. The most frequent clinical presentations are hematemesis, melena and hematochezia [2–5]. When associated with blood depletion and/or difficulty in obtaining peripheral venous access, GIB can underlie a medical emergency, hindering resuscitation procedures [6].

The mortality rate ranges from 5 to 21%, and is strictly linked to the presence of an underlying pathology (such as vascular malformations, hepatopathy, portal hypertension, etc.) [2]. Effective management of the underlying condition and adherence to established protocols and guidelines can significantly reduce this rate. In 80% of cases, GIBs are self-limiting; however, certain factors can influence the prognosis, including the initial approach and the monitoring of the acute phase to identify patients at risk of hemodynamic instability [7–9].

The primary objectives of GIB management are to decrease mortality rates and minimize the necessity for extensive surgical interventions. A secondary aim is to prevent unnecessary hospital admissions for patients experiencing minor or self-limiting bleeding [2].

This review aims to propose a systematic approach for the differential diagnosis of GIB based on its clinical presentation. It aims to underscore key clinical indicators that can help clinicians attain an accurate diagnosis. Additionally, it intends to explore the current indications, advantages, and limitations of available diagnostic procedures.

### Definitions

Hematemesis: emission of bright red blood from the mouth, in case of active bleeding, or “coffee- ground” colored material, in case of non-recent bleeding.

Melena: black and foul-smelling stool emission from the anus. These characteristics are due to the hemoglobin oxidation to hematin by intestinal enzymes and floral bacteria.

Hematochezia: passage of bright red or dark (due to the presence of clots) blood via rectum, isolated or mixed to stool or mucus.

Overt bleeding: passage of visible blood whose origin has not been identified by endoscopic or radiological investigations.

Occult bleeding: passage of not visible blood suggested by laboratory tests (e.g. iron deficiency anemia) and confirmed by positive fecal occult blood test.

Massive bleeding: Gastrointestinal bleeding that results in hemodynamic instability, signs of poor perfusion (e.g. altered mental status, syncope, pallor), need for transfusion of more than 20 ml/kg of packed red blood cells (pRBCs) during the initial resuscitation, or blood loss of more than 80 ml/kg in 24 h, more than 40 ml/kg in 3 h or more than 3 ml/kg/min [7, 10–12].

### Clinical evaluation of child with suspect GIB

When assessing a child suspected of having GIB, the foremost priority is to achieve and maintain hemodynamic stability. Subsequently, inquiring into medical history in detail and performing comprehensive physical examination are crucial steps in the evaluation process.

#### Is it blood?

It is known that many substances, when mixed to vomit or stools, might be confused for bright red blood (such as food coloring contained in jellies, beverages or candies, tomatoes peel, beets and some antibiotic syrups) or melena (drugs containing bismuth or iron, spinach, blueberries, grape or licorice) [3, 10, 11, 13].

Different tests aiming to identify the presence of blood in stools and vomit are nowadays available.

For instance, the Guaiac Test can easily detect blood: it implies the placement of the sample on a guaiac sheet (which contains a phenolic compound, alpha-guaiaconic acid, extracted from Gaiacum trees) and the addition of hydrogen peroxide that, in presence of blood, can oxide guaiac causing a color change in blue [14, 15]. False positive results can occur in case of dietary interference, e.g. red meat containing myoglobin or certain uncooked vegetables containing specific compounds with peroxidase activities. Similarly, Guaiac Test is also subject to negative interference when testing foods containing vitamin C, such as citrus fruits, as their antioxidant properties can inhibit the color reaction used in the testing process, thus causing false negative results [10, 15].

Therefore, some immunochemical tests capable of detecting only human blood (largely used amongst adults as a screening test for colon cancer) were proposed in the pediatric population in order to improve sensibility and specificity in detecting blood in stools. Even though these tests are considered as a gold standard to confirm the presence of blood in red or tarry intestinal secretions, their high sensibility might be a limitation in pediatric patients: indeed, in children fissures and perianal dermatitis are very common and might cause false positive results leading to further unnecessary diagnostic procedures [10, 11]. Therefore, these tests’ results should be wisely evaluated.

#### Is the blood coming from the gastrointestinal tract?

It is extremely important to investigate the presence of either digestive or non- digestive signs and symptoms, such as abdominal pain, vomiting, cough, odynophagia, and fever. Furthermore, a complete and well-directed anamnesis, inquiring into past medical history, can be diriment. For instance, a medical history revealing recent tonsillectomy, dental procedure, epistaxis, or nasogastric tube placement, may suggest oral/nasal bleeding [10, 11].

Furthermore, in physical examination, the clinician should evaluate for signs of gingival traumas and active bleeding coming from oral, nasal, or genitourinary sites [11].

When gathered, this information can help the clinician to avoid common mistakes, as to confuse hemoptysis with hematemesis, or menstruation with rectal bleeding (particularly in adolescents experiencing menarche) [16].

#### What is the entity of bleeding?

The extent of bleeding can be ascertained by assessing the patient's overall appearance and hemodynamic condition.

“Red flags” in signs and symptoms are paleness, diaphoresis, restlessness, lethargy, and abdominal pain. The association of both hematemesis and melena should raise suspicion of an active severe proximal bleeding [6, 10, 13, 16].

Parameters should always be monitored, and they represent a crucial first step in order to evaluate the patient; thus, children have a major physiological reserve when compared to elderly patients and therefore vital signs could remain normal for a longer time. Indeed, in children, it has been demonstrated that hypotension may not be present until up to 15–30% of the circulating blood volume has been compromised [17]. Hence, the most reliable indicator of significant blood loss is an increase in pulse rate of 20 or more beats per minute (bpm) or a decrease in systolic blood pressure of 10 mmHg or more upon transitioning from a supine to a sitting position [2, 10, 11].

#### Which is the site of bleeding?

Upper GIB includes hemorrhage originating from the esophagus to the ligament of Treitz, beside the duodenojejunal flexure. Lower GIB bleeding is defined as bleeding that originates from a site distal to the ligament of Treitz [18].

Hematemesis is the classic presentation of upper GIB (proximal to the ligament of Treitz), while lower gastrointestinal bleeding (distal to the ligament of Treitz) often presents as bloody diarrhea or the passage of bright red blood mixed with or coating normal stools [13, 19]. However, melena, hematochezia, and dark/occult bleeding can stem from both upper and lower gastrointestinal sources. In fact, melena that generally indicates an upper GIB (esophagus, stomach, duodenum, and proximal jejunum), in immunocompromised patients with slow intestinal transit may also origin from bleeding within the small bowel or colon. Similarly, hematochezia, commonly suggesting bleeding from the distal small bowel or colon, can also result from severe upper digestive tract bleeding due to a cathartic effect from large blood volumes in the intestinal lumen, hastening intestinal transit [10].

In doubtful cases, especially in hemodynamically unstable patients experiencing rectal bleeding suspected to stem from significant upper digestive tract hemorrhage, placing a nasogastric (NG) tube and performing a normal saline lavage may help identify the bleeding site, determine the extent of bleeding, assess ongoing bleeding, and mitigate the risk of inhaling gastric contents or developing hepatic encephalopathy in cirrhotic patients [20]. Nonetheless, it is essential to note that the NG tube's negative predictive value is limited, as it might not detect bleeding from the bulb or duodenum. Moreover, while NG lavage effectively reduces gastric fluid accumulation, it does not halt the bleeding [7, 10, 11].

### Special considerations in newborn and infants

In neonates and infants younger than 12 months, unique etiologies of GIB exist. Common causes of GIB in an otherwise healthy infant are anal fissures, eosinophilic proctocolitis or food protein-induced allergic proctocolitis (FPIAP) and ingestion of maternal blood from delivery or fissured nipples during breastfeeding [3, 5, 11, 19, 21].

To distinguish between fetal and maternal origin of blood, the Apt-Downey test can be performed. It exploits the different denaturing properties of fetal and maternal hemoglobin in the presence of sodium hydroxide [3, 11, 16, 22]. Blood is mixed with a small amount of sterile water to cause hemolysis of red blood cells, producing free hemoglobin. The sample is then centrifuged, and the supernatant mixed with 1% sodium hydroxide (NaOH). The fluid color, assessed after 2 min, will remain pink in case of fetal hemoglobin, while it will turn yellow- brown in case of adult hemoglobin because the latter one is less stable and will convert to hematin [10, 22].

In a newborn, GIB may be one of the presenting symptoms of a cow's milk protein allergy or an underlying coagulopathy [23]. Bleeding from vitamin K deficiency should be considered in infants with maternal exposure to antiepileptic drugs that affect vitamin K, dysbiosis from antibiotic exposure, cholestasis, short bowel syndrome, or failure to receive perinatal vitamin K prophylaxis [10, 11]. Vitamin K deficiency is easily corrected by the intramuscular or intravenous administration of vitamin K. Failure to correct bleeding should raise suspicion for congenital bleeding disorders, such as clotting factor deficiencies or von Willebrand’s disease [24].

However, in clinically unstable, premature, or very low birth weight infants, necrotizing enterocolitis should always be suspected. In addition, in this subgroup of infants, severe hematochezia is a late clinical sign in many surgical emergencies, from volvulus to intussusception [11].

In healthy infants younger than 9 months old straining and crying for at least 10 min before successful or unsuccessful passage of soft stools without blood, infant dyschezia should be suspected and parents should be reassured about the benign nature of this condition [25].

### Laboratory tests

Complete blood count and red blood cell indices can shed light on the severity and chronicity of bleeding. A low mean corpuscular volume (MCV) suggests long-duration bleeding even if bleeding has recently arisen [10]. Hemoglobin (Hb) and hematocrit determination are part of the standard procedure, even though initial Hb may be normal [6]. Thrombocytopenia may indicate hypersplenism or, when associated with direct hyperbilirubinemia and increased creatinine levels, uremic-hemolytic syndrome; conversely, thrombocytosis is often associated with inflammatory condition (e.g. chronic inflammatory bowel disease (IBD), subacute infectious enterocolitis…). In case of severe bleeding, changes in serial blood counts may presage a worsening clinical course and the need for therapeutic interventions.

Liver enzymes [alanine aminotransferase (ALT), aspartate aminotransferase (AST), gamma-glutamyl transferase (GGT)], total and fractionated bilirubin, and albumin are used to assess liver function. The coagulation profile [prothrombin time (PT), partial thromboplastin time (PTT) and international normalized ratio (INR)] may indicate pre-existing coagulopathy, chronic hepatopathy, or acute conditions such as sepsis and disseminated intravascular coagulation [3, 16].

An increase in blood urea nitrogen levels may be related to amino acid catabolism during intestinal digestion of red blood cells, and in children, an azotemia/creatinine ratio > or equal to 30 has a sensitivity of 68.8% and specificity of 98% in determining the upper origin of bleeding [3].

Blood typing and crossmatching should always be required in case the patient needs blood transfusion.

In patients with lower intestinal bleeding associated with symptoms of colitis, in addition to blood tests, stool analysis for infectious agents (*Salmonella, Shigella, Clostridium difficile* toxin A and B, *Yersinia, Campylobacter, Entamoeba histolytica* in case of recent travel to a high-risk geographic area, and *Escherichia coli O157:H7* in case of impaired renal function) can be performed based on clinical suspicion (e.g. antibiotic exposure, immunodepression, recent travel) [10, 11].

Additional tests may be pursued according to clinical history, such as inflammatory markers (C-Reactive Protein—CRP) and quantitative fecal calprotectin in suspected IBD. Quantitative fecal calprotectin is a marker for intestinal inflammation with high negative predictive value. Its determination is useful to support the diagnosis of IBD in patients with hematochezia associated with colitis symptoms for more than 2 weeks, but false positive results can be obtained throughout NSAIDs and proton pump inhibitors therapy [2, 10].

## Upper gastrointestinal bleeding

### Hematemesis

Table 1 provides an overview of common and rare causes of hematemesis based on age, onset, and bleeding characteristics.

**Table 1:** Common and rare causes of upper gastrointestinal bleeding according to the age, appearing and bleeding entity

	**Ill-appearing**	**Well-appearing**	
		**Severe bleeding**	**Milder bleeding**
** < 2 years**	Stress gastritis or ulcer Sepsis *Rare:* Intestinal duplications Vascular anomalies Coagulation disorders		Reflux esophagitis Reactive gastritis Vitamin K deficiency Trauma (NG tube)^a^ *Rare:* Cow’s milk protein allergy
**2–5 years**	Esophageal varices Hemorrhagic gastritis Stress ulcer	Esophageal varices Gastroduodenal ulcer Foreign bodies Ingestion of caustics *Rare:* Dieulafoy lesion Arteriovenous malformations Stromal tumors Gastroduodenal duplications	Mallory-Weiss tear NSAIDs^b^ gastritis Reflux esophagitis
** > 5 years**	Esophageal varices Hemorrhagic gastritis	Esophageal varices Gastroduodenal ulcer *Rare:* Dieulafoy lesion Arteriovenous malformations Stromal tumors Gastroduodenal duplications Hemobilia	Mallory-Weiss tear Reflux esophagitis Gastritis

^a^*NG* Nasogastric tube

^b^*NSAIDs* Non Steroid Anti-inflammatory Drugs

A chronic history of symptoms such as heartburn, regurgitation, epigastric pain, or difficulty in swallowing should raise suspicion of reflux esophagitis or peptic ulcer disease. Persistent vomiting is frequently observed during acute episodes of cyclic vomiting, in infants with hypertrophic pyloric stenosis, or in cases of acute gastroenteritis. However, this symptom can also indicate acute erosive esophagitis or a Mallory-Weiss laceration (an acute mucosal tear at the esophagogastric junction) [26].

Reactive gastritis and ulcers with substantial bleeding can be associated with polytrauma, surgical procedures lasting longer than three hours, and recovery in the intensive care unit, particularly when linked with sepsis and lung failure, especially in cases requiring mechanical ventilation.

The use of NSAIDs and Helicobacter pylori represent two other significant risk factors for gastritis and ulcer development [3, 7, 13, 27].

Hematemesis can be the first manifestation of esophageal varices. Bleeding from varices should be suspected in children with a medical history of chronic liver disease, cystic fibrosis, right heart failure or conditions associated with extrahepatic portal thrombosis (history of abdominal surgery or neonatal sepsis, omphalitis, blood transfusion and umbilical vein catheterization) and in case of hepatosplenomegaly, ascites or jaundice observed during physical examination [3].

Rarely, hematemesis can be the result of submucosal masses that, eroding mucosa, can bleed (stromal tumors, gastroduodenal duplications), or hemangiomas and Dieulafoy lesions (aberrant submucosal artery protruding through a minute defect in the mucosa, provoking a massive bleeding) [10, 22, 28, 29].

Furthermore, recurrent cough or acute dysphagia and abdominal pain may suggest unwitnessed foreign body ingestion, thus chest and abdomen x-rays are required [19]. Children who presented with hematemesis but appear in good general conditions, and in which routine exams have turned negative, can be discharged with prescription of oral proton pump inhibitors and followed as outpatients. Conversely, children younger than one year old or children who presented with significant bleeding or physical or biochemical findings suggestive of portal hypertension should be hospitalized to undergo esophagogastroduodenoscopy (EGDS) [10, 22]. At the same time, all neonates with hematemesis should be screened for coagulopathy due to vitamin K deficiency, maternal thrombocytopenic purpura, hemophilia, and von Willebrand disease [10].

**Fig. 1:**
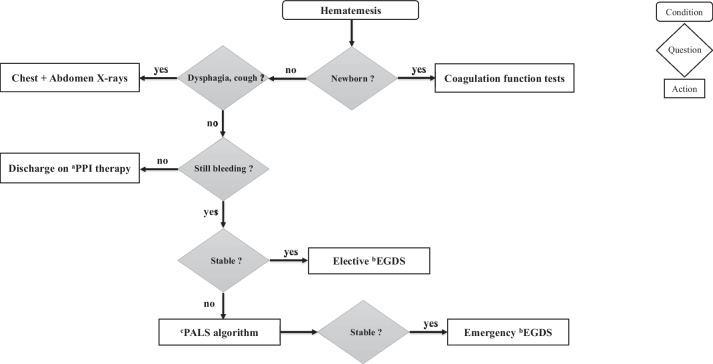
Proposed algorithm for the approach to children with hematemesis. Legend: ^a^PPI: Proton Pump Inhibitors; ^b^EGDS: Esophagogastroduodenoscopy; ^c^PALS: Pediatric Advanced Life Support

EGDS is the gold standard procedure to evaluate children with hematemesis [2]. It aims to identify sources of bleeding, diagnose the underlying cause, assess bleeding risk, and potentially administer endoscopic therapy [22]. Emergency EGDS (as soon as the patient is stable) is necessary only if the bleeding persists and is associated with hemodynamic instability that does not respond to blood transfusion. In such cases, it is preferable to perform the procedure under general anesthesia and orotracheal intubation, to ensure better airway control. In other cases, EGDS can be performed within the 24–48 h from the beginning of the symptom [5, 7, 11, 13, 21, 22]. Elective EGDS is recommended for patients with massive or recurrent hematemesis, unexplained iron-deficiency anemia, persistence of acid-related symptoms after suspension of acid-suppressive therapy, or in cases with suspected portal hypertension indicated by clinical-laboratory signs of liver disease or hypersplenism (thrombocytopenia and leukopenia) [2, 10, 22]. Figure 1 outlines an algorithm guiding the approach to children with hematemesis.

Mortality rate for hematemesis in children ranges from 5 to 15%, given that incidence of conditions associated with upper gastrointestinal bleeding (UGIB), such as acute variceal hemorrhage, widely differs within Western and Eastern countries [2].

## Lower gastrointestinal bleeding

In Table 2 common and rare causes of lower gastrointestinal bleeding (LGIB) according to the age, appearing and bleeding entity have been listed.

**Table 2:** Common and rare causes of lower gastrointestinal bleeding according to the age, appearing and bleeding entity

	**Ill-appearing**	**Well-appearing**	
		**Severe bleeding**	**Milder bleeding**
** < 2 years**	Intussusception Volvulus Infective colitis *Rare:* Necrotizing enterocolitis Hirschsprung enterocolitis Vascular malformation		Anal fissures Allergic proctocolitis Lymphoid hyperplasia Infective colitis
**2–5 years**	Intussusception Volvulus Henoch-Schönlein purpura Uremic-hemolytic syndrome	Meckel diverticulum Esophageal varices Ulcerative colitis *Rare:* Juvenile polyp Radiation enterocolitis Neutropenia associated colitis Vascular malformation	Infective colitis Juvenile polyp Lymphoid hyperplasia Ulcerative colitis Perianal streptococcal cellulitis *Rare:* Rectal prolapse/ulcer Crohn’s disease
** > 5 years**	Infective colitis Ulcerative colitis Henoch-Schönlein purpura Volvulus/intussusception	Ulcerative colitis Meckel diverticulum Esophageal varices *Rare:* Vascular malformation	Infective colitis Ulcerative colitis Juvenile polyp Hemorrhoids NSAIDs^a^ *Rare:* Rectal prolapse/ulcer Crohn’s disease

^a^*NSAIDs* Non steroid anti-inflammatory drugs

It is important to emphasize that intestinal bleeding can manifest as an epiphenomenon within various clinical conditions. From this perspective, it is essential to explore and consider all the signs and symptoms that relate to the phenomenon of intestinal bleeding encountered.

### Melena and hematochezia

About 10 to 15% of mucosal or variceal hemorrhages from the upper GI tract may present with melena alone or seldom with passage of gross bright red blood through rectum without hematemesis [10].

Moderate-severe hematochezia in a child with abdominal pain, especially when ill-appearing, might hint intestinal ischemia secondary to intussusception or volvulus. Idiopathic intussusception is more common in children younger than 2 years old and occurs mostly in children with a recent history of viral infection. In this condition the most common symptoms are vomiting (70.8%) and abdominal pain (60.6%).

In patients aged younger than 1 year, “currant jelly” bloody stool, abnormal abdominal radiography findings, and a longer intussusceptum segment are more frequent [16, 28, 30].

In children older than 2 years old, invagination is more likely to be associated with a lead point as a Meckel diverticulum, polyps, lymphoid hyperplasia, intestinal duplication, lymphomas, intestinal wall edema (as in Henoch-Schӧnlein purpura). It is noteworthy to mention that in 15–25% of children affected by Henoch-Schӧnlein purpura, GIB can precede cutaneous manifestations by even a week [2, 10, 16, 19].

Moderate and severe melena and hematochezia without abdominal pain can suggest the presence of Meckel diverticulum, a vascular malformation (i.e. angiodysplasia, Dieulafoy lesion) and less often an autoamputation of a juvenile polyp [29, 31]. In the latter case, parents often report finding tissue fragments in blood. It is also noteworthy to mention that NSAIDs may cause ulcerations in the ileum and the colon [11, 13, 19, 27, 28].

Abdominal ultrasonography, if necessary integrated by abdominal computed tomography (CT), is the first-line option to exclude surgical causes (e.g. intussusception, volvulus, masses) in ill-appearing patients with lower GIB and abdominal pain [32]. Ultrasonography (US) is relatively inexpensive, widely available, does not involve ionizing radiation and can provide dynamic assessment of bowel vascularization (Doppler) and peristalsis [33, 34]. The main limitation of this diagnostic instrument is that intense intestinal meteorism can reduce its sensibility. CT can provide panoramic and standardized abdominal imaging in a short time, remaining the gold standard in case of emergency/urgency. Indeed, abdomen plain film x-rays are not recommended in case of emergency/urgency, having proved lower diagnostic accuracies than abdominal CT scans [35]. In performing CT, the administration of an intravenous contrast medium is essential [36].

A pathognomonic sign of the volvulus is the so-called “Whirlpool Sign”, a spiraling of the superior mesenteric vein around the superior mesenteric axis, easily recognizable on CT [36].

Intestinal intussusception may present with different ultrasound patterns, the most common being the so-called “doughnut” or “target” on the transverse scans, appearing as an oval hypo-echoic mass with bright central echoes [33, 37]. Transversal scans can also reveal a peculiar “concentric rings” structure inside the mass, also called “complex mass”, depending on the degree of bowel wall edema [33, 37]. When the intussusception is caused by a lead point (expansive lesion), this can be identified as a solid mass inside the bowel loop, both on US and on CT scan. Currently, given its high sensibility and specificity, the diagnosis of intussusception can be performed with US alone, saving the enema (with air or water) only for therapeutic treatment [37, 38].

In children with bleeding and no abdominal pain or inflammatory markers, the immediate step before colonoscopy is to perform a Meckel scan (99Tc-pertechnetate nuclear scan) to look for a Meckel diverticulum [10, 16]. An important limitation of this diagnostic instrument is the low sensitivity (60%) and negative predictive value (76%) [39]. The most common cause of a false positive result is gastrointestinal duplication, since it contains internal gastric heterotopic mucosa that can concentrate the Tc-pertechnetate [16, 40, 41]. Vascular malformation, GIB not related to ectopic gastric mucosa and tracer concentration by genito-urinary system can be associated with false positive results, wrongly diagnosed as Meckel diverticulum. False negative results can also occur from small amounts of ectopic gastric mucosa in the diverticulum or technical problem [16, 40–42]. Premedication with H2 blockers and delayed imaging can increase diagnostic accuracy of the procedure [11, 28, 41, 42].

Single Photon Emission Computed Tomography (SPECT) or SPECT-CT can overcome Meckel scan limitations, increasing the sensitivity and the specificity of the procedure. Indeed, SPECT can increase contrast resolution over conventional planar imaging and hence the ability to detect even smaller volumes of GIB. This technique can also help distinguish vascular from musculocutaneous lesions and differentiate vascular variants from true obscure gastrointestinal bleeding (OGIB), providing better anatomical details. However, SPECT entails an increased radiation burden and may be reserved to adults where differential diagnoses may occur simultaneously [43, 44].

After excluding Meckel diverticulum and/or surgical causes, upper endoscopy and colonoscopy are the procedures of choice to achieve etiological diagnosis and, in case of polyps and vascular malformation, to perform therapeutic endoscopy [10, 36]. Before the colonoscopy, a fast bowel cleansing administered within a nasogastric probe is highly recommended to improve diagnostic yield, reduce the risks and facilitate potential therapeutic performances [2, 20].

In cases of persistent bleeding and endoscopic failure to identify the source of bleeding, multiphasic dynamic computed tomography with specific flow rate timing is recommended. CT angiography, particularly in cases of active bleeding, demonstrated a sensitivity of 70–90% and specificity of 99–100%. This imaging technique can identify active bleeding but also pinpoint its source, providing valuable guidance for therapeutic interventions. The advantages of CT include rapid acquisition within a few minutes and the ability to detect active bleeding as low as 0.3–0.5 ml/min [10, 32, 36].

**Fig. 2:**
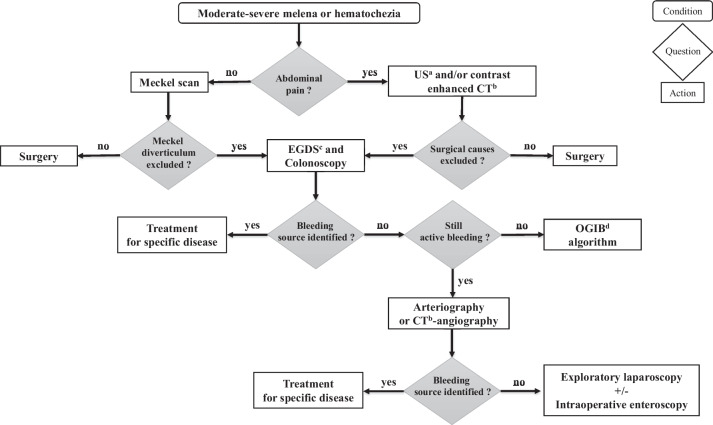
Proposed algorithm for the approach to children with moderate-severe melena or hematochezia. Legend: ^a^US: Ultrasonography; ^b^CT: Computed Tomography; ^c^EGDS: Esophagogastroduodenoscopy; ^d^OGIB: Obscure Gastrointestinal Bleeding

As a result, traditional arteriography primarily serves a therapeutic role through selective or superselective catheterization. It enables embolization of the bleeding lesion or placement of markers inside or nearby the bleeding area, facilitating localization during subsequent surgery [8, 16]. However, the therapeutic potential of arteriography is limited to arterial sources of bleeding and carries a high risk of femoral artery thrombosis and intestinal ischemia [16]. When the bleeding persists and the procedures previously described did not identify the lesion, exploratory laparoscopy, combined when needed with intraoperative enteroscopy, represents the suitable procedure to both identify and treat the cause of bleeding [10, 36]. In Fig. 2 the algorithm for the approach to children with melena or moderate-severe hematochezia is outlined.

**Fig. 3:**
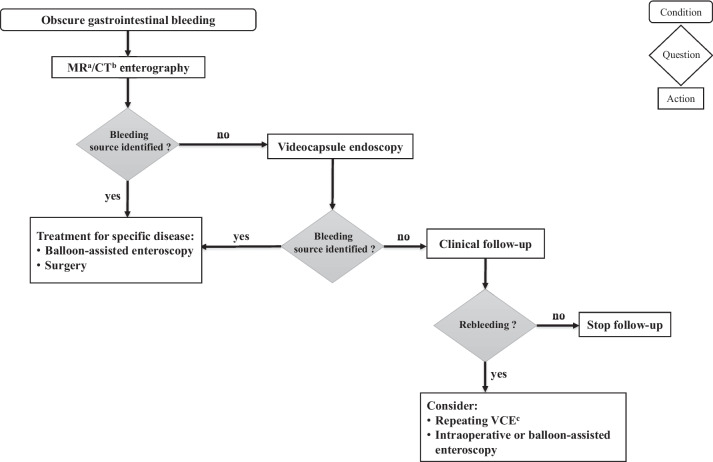
Proposed algorithm for the approach to children with obscure gastrointestinal bleeding. Legend: ^a^MR: Magnetic Resonance; ^b^CT: Computed Tomography; ^c^VCE: Videocapsule endoscopy

For patients with resolved bleeding and inconclusive findings on endoscopy (obscure bleeding), MRI (Magnetic Resonance Imaging) enterography, capsule endoscopy, and enteroscopy are key diagnostic approaches. MR/CT enterography can reveal abdominal wall defects (e.g. Meckel diverticulum, intestinal duplication and polyps), while capsule endoscopy demonstrates a high diagnostic yield for mucosal lesions, especially small vascular abnormalities during active bleeding [34, 36]. The main limitations of capsule endoscopy are the risk for retention (that can be prevented by a prior investigation through small bowel imaging) and the impossibility to control progression of the capsule with consequent low diagnostic yield in case of high peristaltic contraction [11]. Owing to the latter one, in case of persistent bleeding, another evaluation through capsule endoscopy as near as possible to the bleeding source and an improved bowel cleansing could become necessary [34, 36, 45]. Balloon-assisted enteroscopy or intraoperative enteroscopy may help classify and treat lesions detected by MR/enteroscopy or by capsule endoscopy [28, 32, 46, 47]. Figure 3 outlines an algorithm guiding the approach to children with OGIB.

The mortality of LGIB bleeding is estimated around 0.9% [48].

### Rectal bleeding with signs of colitis

Signs of colitis are bloody diarrhea, tenesmus, urgence to defecate, nighttime stooling and abdominal pain.

In a well infant younger than 6 months of age, infective colitis and allergic proctocolitis are the most frequent causes of acutely bloody stools. Oppositely, in a same age ill-appearing infant, late-onset necrotizing enterocolitis and Hirschsprung disease-associated enterocolitis must be suspected. Especially in case of a previous history of chronic constipation dating to early infancy with delayed emission of meconium, Hirschsprung disease should be carefully investigated [2, 10, 11].

In older children (aged more than 2 years), the most common causes of bloody stools are infective colitis that sometimes can be associated to haemolytic-uremic syndrome (e.g. *Escherichia coli O157:H7*, some species of *Shigella dysenteriae*) and IBD, especially ulcerative colitis. In 70% of children who develop haemolytic-uremic syndrome, bloody diarrhea precedes the recognition of haemolytic anemia, thrombocytopenia, and renal insufficiency by 3 to 16 days [49]. Whereas, the presence of arthralgia or arthritis and weight loss support the hypothesis of IBD.

Radiation colitis should be considered in oncology patients treated with radiotherapy. Similarly, neutropenia associated colitis can be observed in patients with leukemia treated with cytotoxic drugs or in other forms of myelosuppression [10, 11].

Laboratory tests and stool culture, as previously detailed in the specific paragraph, should always be obtained. In adolescents who present with perianal secretions, a perianal culture for *Neisseria gonorrhea* should be required. In an immunocompromised patient, searching for Cytomegalovirus (CMV) on stools and, if needed, on biopsies should be considered [10, 28, 50].

Colonoscopy is indicated for patients with clinical or laboratory evidence of chronic inflammation (more than 5 bloody stools per day, nighttime stooling, anemia, hypoalbuminemia) or in well-appearing patients with persistent bloody diarrhea for more than two weeks or with fecal calprotectin high levels [7, 10].

### Rectal bleeding in which blood is mixed with normal-appearing stool

In an otherwise healthy infant under 6 months of age, the presence of blood mixed with normal-appearing stool may suggest conditions such as eosinophilic proctocolitis or nodular lymphoid hyperplasia. Conversely, in children over 2 years of age, blood in the stool is more likely associated with colonic polyps rather than nodular lymphoid hyperplasia [51].

Colonoscopy is recommended for any child experiencing persistent bleeding that cannot be attributed to anal causes. It is also indicated for children with anemia or evidence of positive occult blood/calprotectin between episodes of rectal bleeding [2].

### Rectal bleeding in which blood coats a normal-appearing or hard stool

Rectal bleeding with blood coating the stool often indicates perianal disease, especially when associated with symptoms like anal pain or dyschezia. Anal fissures are frequent in infants younger than 1 year of age, often linked to a history of constipation or recent acute diarrhea. In cases featuring perianal erythema and anal fissures accompanied by secretions, it is essential to rule out streptococcal cellulitis by conducting an anal canal culture.

In older children, recurrent anal fissures should raise suspicion of sexual abuse or, in case of specific lesions like skin tags, Crohn’s Disease.

Solitary rectal ulcers are uncommon in children and are typically linked to constipation and excessive straining during bowel movements [52]. This strain can result in the prolapse of rectal mucosa into the anal canal, leading to congestion, edema, and ulceration [2, 8].

External hemorrhoids are rarely causes of bleeding, unless irritated by excessive cleaning after defecation.

In patients with these symptoms, colonoscopy is needed only in case of persistent bleeding and, in this instance, retroflexion maneuver in rectum is fundamental to better evaluate the internal anal region [10].

## Predisposing conditions to take into consideration in the approach to gastrointestinal bleeding

A previous bowel resection might increase the risk of having a bleeding ulcer from anastomosis. Furthermore, bleeding risk could be higher in case of medication history revealing the use of non-steroid anti-inflammatory drugs (NSAIDs) or anticoagulants in the last month [3, 53]. It is also known that a higher mortality rate due to GIB is associated with the administration of corticosteroids to newborns. Moreover, mucosal tears, ulcerations or life-threatening aortoenteric fistulae might be provoked by the ingestion of disc batteries or sharp objects [11]. In children who underwent recent surgery (from few hours to several days), no personal or familiar history of bleeding, who suffer from continuous bleeding, rarer causes of GIB like Surgery-Associated Acquired Hemophilia A (SAHA) must be suspected and coagulation functions must be assessed [54].

During the head and neck examination, the clinician should search for pigmented macules (freckles) on the lips or buccal mucosa, typically observed in Peutz-Jeghers Syndrome, in addition to scleral icterus and conjunctival pallor [11].

The abdominal examination should evaluate for distension, tenderness to palpation, hepatosplenomegaly, and other stigmata of chronic liver disease (such as ascites, prominent abdominal veins) [26]. Anal inspection may reveal the presence of anal skin tags or perianal fistulae suspicious for Crohn's disease, hemorrhoids, or fissures; rectal exploration may also identify rectum polyps [10, 11, 26].

Skin findings that may raise suspicion for underlying chronic illness are hematomas, who may be found in coagulopathies, and telangiectasias, bluish nodules, and hemangioma, that can be clue for multisystem vascular diseases such as hereditary hemorrhagic telangiectasia, blue nevus syndrome, and visceral cutaneous angiomatosis with thrombocytopenia [11].

## Conclusion

In conclusion, managing GIB in children requires a systematic approach to achieve optimal outcomes. Initial priorities include stabilizing the patient's hemodynamic status, followed by a comprehensive clinical assessment and appropriate diagnostic procedures. Accurately discerning true blood from other substances that might mimic bleeding and identifying the location and severity of the bleed are crucial. Various laboratory tests can provide valuable insights into the chronicity and underlying causes of the bleeding. For newborns and infants, unique considerations, such as maternal–fetal blood differentiation, should be taken into account. The ultimate goal of GIB management is to reduce mortality rate and the necessity for major surgical interventions while minimizing unnecessary hospital admissions for minor or self-limiting bleeding cases. Adhering to established protocols and guidelines, and providing specialized care, significantly enhances outcomes for children grappling with GIB.

## Data Availability

Not applicable.

## Abbreviations

GIB: Gastrointestinal bleeding
GI: Gastrointestinal
LGIB: Lower gatrointestinal bleeding
UGIB: Upper gastrointestinal bleeding
OGIB: Obscure gastrointestinal bleeding
NSAIDs: Non-steroid anti-inflammatory drugs
NG: Naso-gastric
MCV: Mean corpuscular volume
Hb: Hemoglobin
IBD: Inflammatory bowel disease
ALT: Alanine aminotransferase
AST: Aspartate aminotransferase
Gamma-GT: Gamma-glutamyl transferase
PT: Prothrombin time
PTT: Partial thromboplastin time
INR: International normalised ratio
CRP: C-reactive protein
EGDS: Esophagogastroduodenoscopy
CT: Computed tomography
MR: Magnetic resonance
US: Ultrasonography
CMV: Cytomegalovirus
SAHA: Surgery-associated acquired hemophilia A
